# Topography of the frontal branch of the facial nerve and its clinical implication for temporal direct browplasty

**DOI:** 10.1038/s41598-023-40206-5

**Published:** 2023-08-31

**Authors:** Kang-Jae Shin, Shin-Hyo Lee, Young-Chun Gil, Hyun Jin Shin

**Affiliations:** 1 Department of Anatomy and Cell Biology, Dong-A University College of Medicine, Busan, Republic of Korea; 2 Department of Anatomy, Wonkwang University School of Medicine, Iksan, Republic of Korea; 3 Department of Anatomy, College of Medicine, Chungbuk National University, Cheongju, Republic of Korea; 4 Department of Ophthalmology, Research Institute of Medical Science, Konkuk University Medical Center, Konkuk University School of Medicine, 120 Neungdong-ro, Gwangjin-gu, Seoul, 05030 Republic of Korea

**Keywords:** Anatomy, Eyelid diseases

## Abstract

Due to anatomic proximity to the surgical site, iatrogenic trauma to the frontal branch of the facial nerve (FbFN) with resultant brow paralysis is a recognized major complication of temporal direct browplasty. This study was aimed to elucidate the course of the FbFN in the area superolateral to the brow in order to facilitate safer temporal direct browplasty by preventing facial nerve injury. Forty-five hemifaces from 32 embalmed Korean cadavers were dissected. A horizontal line connecting the tragion to lateral canthus was established. Then, an oblique line passing through the lateral canthus and 45° to the horizontal line was used as reference line. The mean distance from the lateral canthus to the points where the FbFN cross the reference line was measured. The angle between the FbFN and reference line at the crossing points were also recorded. After crossing the zygomatic arch, FbFN continues in an anteriorly inclining curve across the temporal region, passing near the lateral end of the brow as it heads toward frontalis muscles. During the course, the FbFN laying in the innominate fascial layer was divided into 3 branches. The anterior and posterior branch of FbFN crossed the reference line superiorly and laterally at 3 and 4 cm from the lateral canthus, respectively. In conclusion, the oculofacial surgeon must bring the dissection plane of the forehead tissue more superficially around the 3 cm superolaterally to the lateral canthus in the direction of 45° from the horizontal line in order to avoid nerve injury.

## Introduction

Brow ptosis is decent of the brow and brow fat pad and typically occurs with advancing age or prior trauma, or facial nerve palsy. It can affect patients' quality of life because of cosmetically displeasing appearance, or visual field impairment as a result of excess soft tissue pushing downwards on the eyelid^1, 2^. Just an upper lid blepharoplasty will not solve this issue. Repeated upper lid blepharoplasty attempts to treat a ptotic brow will fail because they will descend the brow even farther. The surgical technique must consequently include the independent correction of the ptotic brow^3^.

Direct browplasty, also known as a brow lift, is a surgical procedure designed to raise and reshape the eyebrow region by removing excess skin, subcutaneous tissue, and muscle from the forehead. This procedure allows the surgeon good control over the amount of tissue removed^4, 5^ Especially, patients with a predominant temporal droop may be treated with temporal direct browplasty with incision placement over the lateral one half or one third of the eyebrow^6^.

The sensory and motor nerves of the forehead must be protected during any procedure to elevate the brows. The supraorbital nerve (SON) provides sensation to the forehead and scalp. It extends vertically from the supraorbital notch toward the crown and is located on the surface of the frontalis muscle in the middle of the forehead^7^. The motor innervation for the forehead muscles comes from the frontal branch of the facial nerve (FbFN). This branch crosses the zygomatic arch and courses deep to the frontalis muscle in the forehead, running horizontally from posterior to anterior^8^.

Previous studies have focused on the course of the SON in the medial forehead area for preventing iatrogenic trauma to the SON with resultant paresthesia or numbness of the forehead, while relatively few studies have examined the course of FbFB in the superotemporal region^9^. However, due to the relatively scant subcutaneous tissue that protects it along its course, the FbFN is one of the nerves most commonly damaged in temporal direct browplasty^2^. Thus, in temporal direct browplasty performed in the lateral of the eyebrow, it is important to remember the path of the FbFN to prevent the possibility of complete brow paralysis^3, 10^.

The medial zygomaticotemporal vein (MZTV) has been observed in the vicinity of the FbFN during endoscopic temporal brow lift and is considered an important consistent landmark for the conservation of the FbFN in the lateral orbital area^11^. Few authors, however, have explained their exact methods of dissection in the temporal regions during the temporal direct browplasty. Some surgeons advocate a superficial dissection, a supra-aponeurotic plane. Gonzales-Ulloa^12^ stated that, "when passing from the forehead to the temporal region, it is important to change from the supra-periosteal level to a supra-aponeurotic one." Liebman et al.^13^ said that the "danger zone" or "red zone," which extends from the frontalis muscle medially to the zygomatic arch laterally, lies within the 2-cm-wide area superolateral to the brow in the superficial musculoaponeurotic system (SMAS) layer.

The purpose of this study is to describe the course of the FbFN in the area of superotemporal to brow in order to facilitate safer approaches and avoid complications during temporal direct browplasty, as well as to define the depth of the FbFN relative to the best plane of dissection in performing the temporal direct browplasty.

## Materials and methods

Sixty-two hemifaces of 32 embalmed adult Korean cadavers were dissected. After excluding 17 hemifaces that had been disrupted anatomically, 45 hemifaces were suitable for morphometric measurements (comprising 22 male and 10 female orbits, and 18 right and 27 left orbits). The mean age at the time of death was 76.3 years (range, 60–95 years). All procedures were performed in accordance with the Declaration of Helsinki of the World Medical Association (WMA). After the approval of the Dong-A University College of Medicine, twenty-five embalmed cadavers that were legally donated to this institution, were subjected to the dissection of the maxillofacial region. The cadavers were not prisoners and informed consent was obtained from the legal guardians.

### Cadaveric dissection

**Figure 1:**
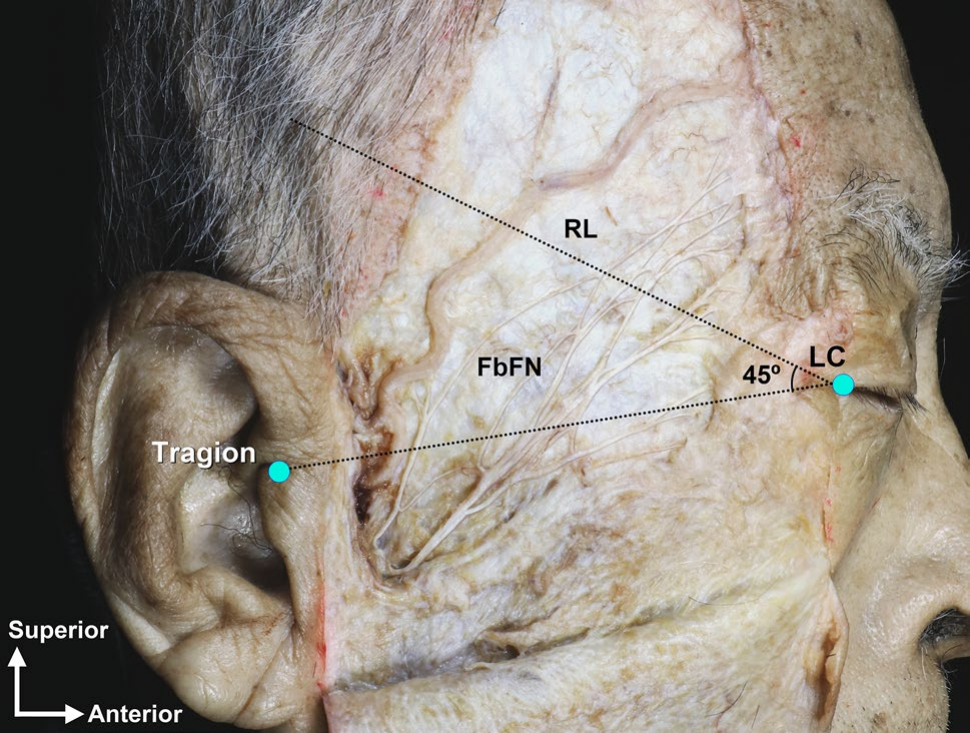
Cadaveric dissection demonstrating the location of the frontal branch of facial nerve (FbFN), reference line (RL), tragion and lateral canthis (LC).

Anatomical dissection was performed via a transcutaneous approach in a layer-by-layer fashion. The skin and subcutaneous tissues were carefully removed, and the main trunk of the facial nerve was identified in the parotid gland. Then, FbFN was meticulously delineated along their courses, and traced forward to the frontalis area^8^. For recording of the location of the FbFN, a horizontal line through the tragion and the lateral canthus was made. Then a reference line (RL) passing through the lateral canthus and 45° to the horizontal was used for determining the crossing points of FbFN (Fig. 1). The FbFN twigs closest and farthest to the lateral canthus on the RL were defined as the anterior branch of FbFN (FbFN-A) and posterior branch of FbFN (FbFN-P), respectively.

### Measurements

The RL was divided by 5 mm intervals from the lateral canthus and the number of FbFN twigs crossing RL in each section were recorded. For simplification, tiny and thread-like finer nerve strands were not considered in this recording. Also, the following parameters (referenced subsequently as #1 to #4) were measured directly on the cadavers using digital calipers (CD-15CPX, Mitutoyo, Kanagawa, Japan):

1. Distance from the lateral canthus to the crossing point of FbFN-A on the RL
2. Angle between the FbFN-A and the RL at the crossing points
3. Distance from the lateral canthus to the crossing point of FbFN-P on the RL
4. Angle between the FbFN-P and the RL at the crossing points

### Statistics

The Statistical Package for the social sciences version 27.0 for Windows software program (IBM Corp., Armonk, NY, USA) was used for statistical analysis. The Shapiro–Wilk test was used to determine whether the data conformed to a parametric (Gaussian) or nonparametric (non-Gaussian) distribution. Full faces were examined in their two halves in the 13 cadavers and the side difference (laterality) was verified using Paired samples-t test and Wilcoxon singed rank test. Statistical significance was set at *p* < 0.05.

## Results

**Figure 2:**
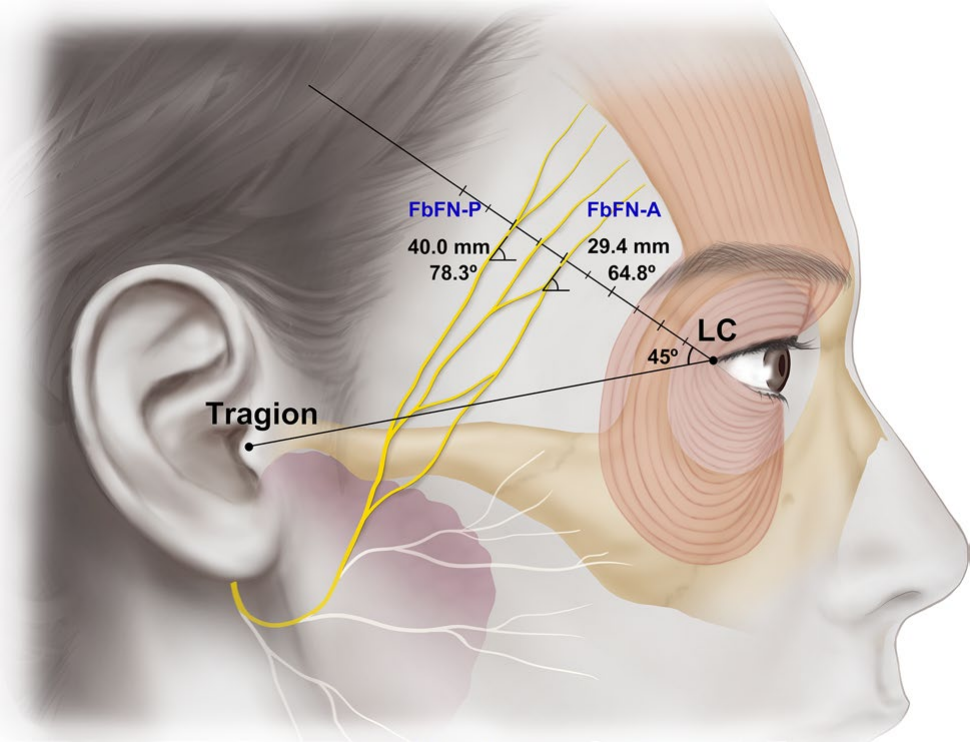
Illustrations of the measured parameters. Each of the numbered measurements is described in detail in the text (Measurements section in the Materials and Methods). FbFN-A, anterior branch of FbFN; FbFN-P, posterior branch of FbFN.

The measurement values are listed in Table 1 and displayed in Fig. 2. “#” is a measurement number of an anatomical picture/diagram. All of the measurements conformed to a normal distribution except #3. There was no laterality difference in the variables between the left and right sides of the hemiface (all *p* > 0.05).

**Table 1:** Measurement values.

Anatomic variable (measurement number)			Mean	SD
Anterior branch of FbFN	Distance	(1)	29.4	4.4
	Angle	(2)	64.8	13.5
Posterior branch of FbFN	Distance	(3)	40.0	6.8
	Angle	(4)	78.3	21.9

The detail descriptions according to numbers are in the text and Fig. 2

Unit: Distance = millimeter, Angle = degree.

*FbFN* Frontal branch of the facial nerve.

**Figure 3:**
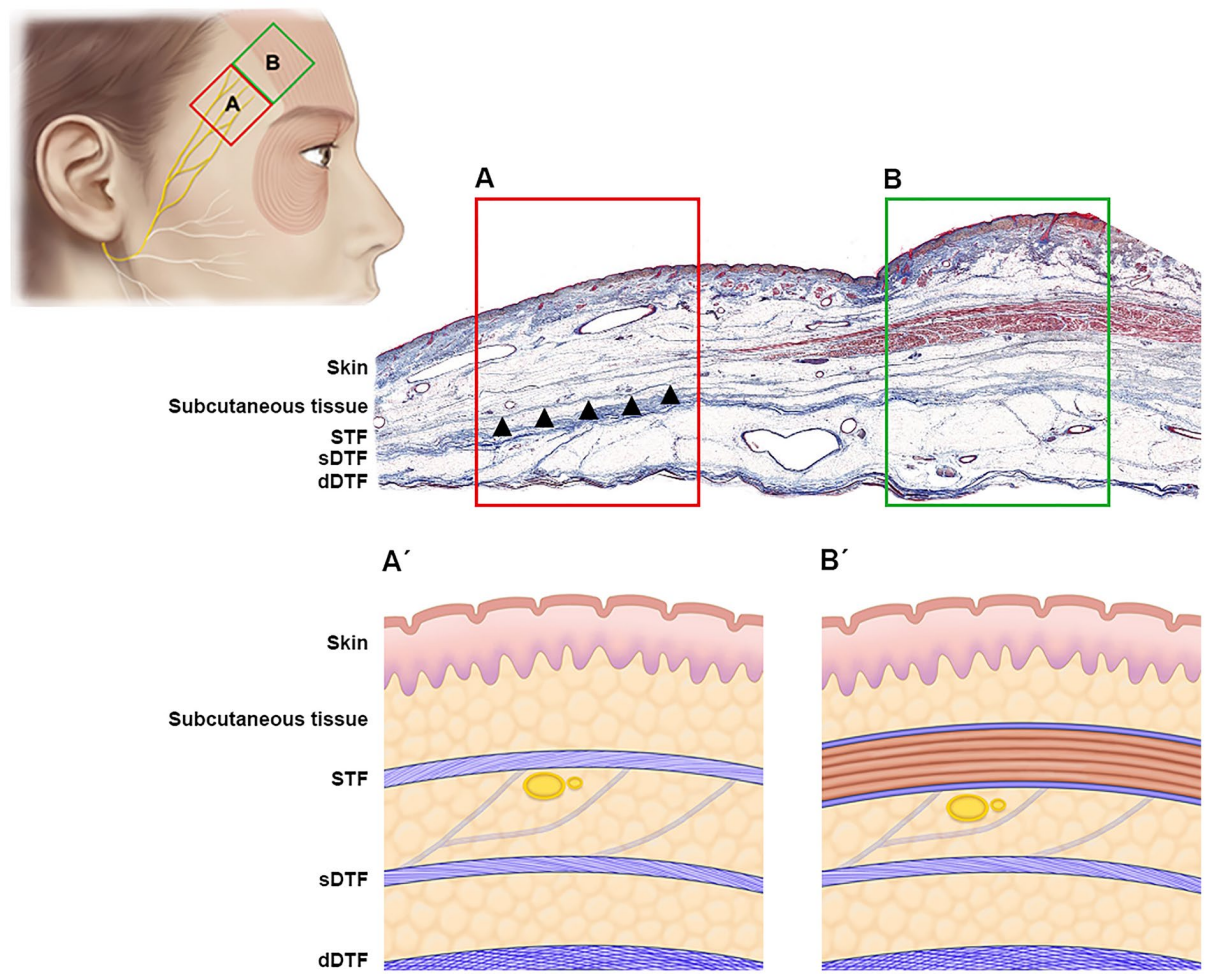
Histologie depth of FbFN at levels (**A**) and (**C**). Danger zone (red box) within which FbFN run in superficial musculoaponeurotic system without muscle cushion.

The FbFN traverses the zygomatic arch then continues in an anteriorly inclining curve across the temporal region, passing near the superior and lateral point of the eyebrow as it heads toward frontalis muscles. The FbFN runs in an intra-innominate fascia plane directly underneath the superficial temporal fascia (Fig. 3).

**Figure 4:**
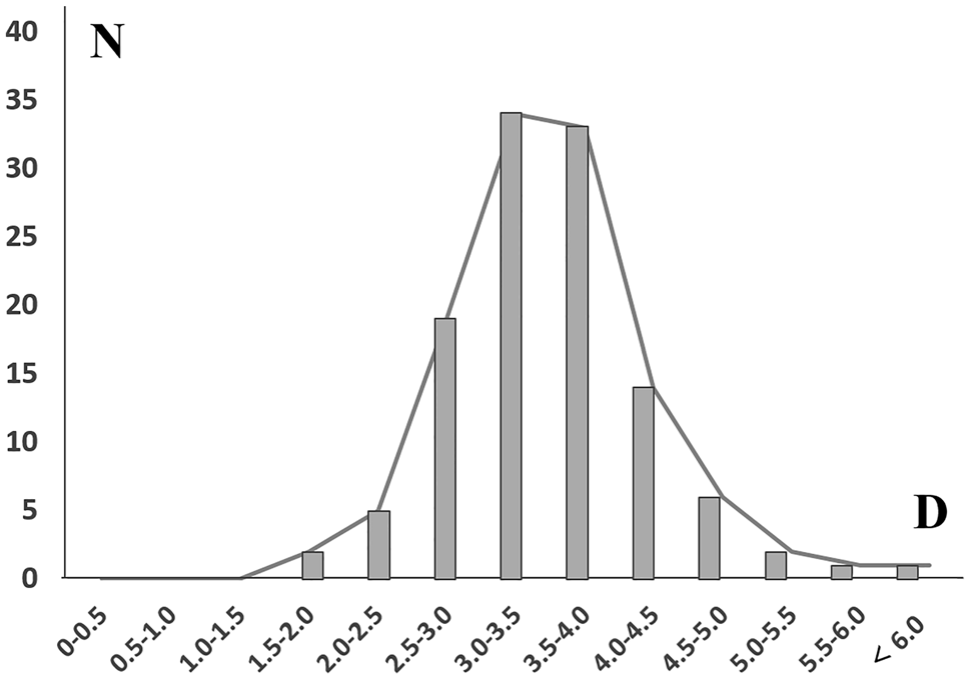
Distribution of FbFN at the level of RL in the temporal region.

The number of FbFN twigs crossing the RL were 3.2 ± 1.1 (range 2–6) with distance range from 18.3 to 61.9 mm from the LC. The mean distance from the lateral canthus to the FbFN-A cossing the RL (#1) was 29.4 mm. The mean distance from the lateral canthus to the FbFN-P crossing the RL (#2) was 40.0 mm. The angle between the FbFN-A and the RL was 64.8° (#3), which was smaller than that of FbFN-P (78.3; #4). The incidence of which FbFN crosses the RL at 3.0–3.5 section from the lateral canthus was predominant (34 specimens, 76%) followed by the 3.5–4.0 mm (33 specimens, 73%) and the 2.5 – 3.0 mm Sect. (19 specimens, 42%) (Fig. 4).

## Discussion

**Figure 5:**
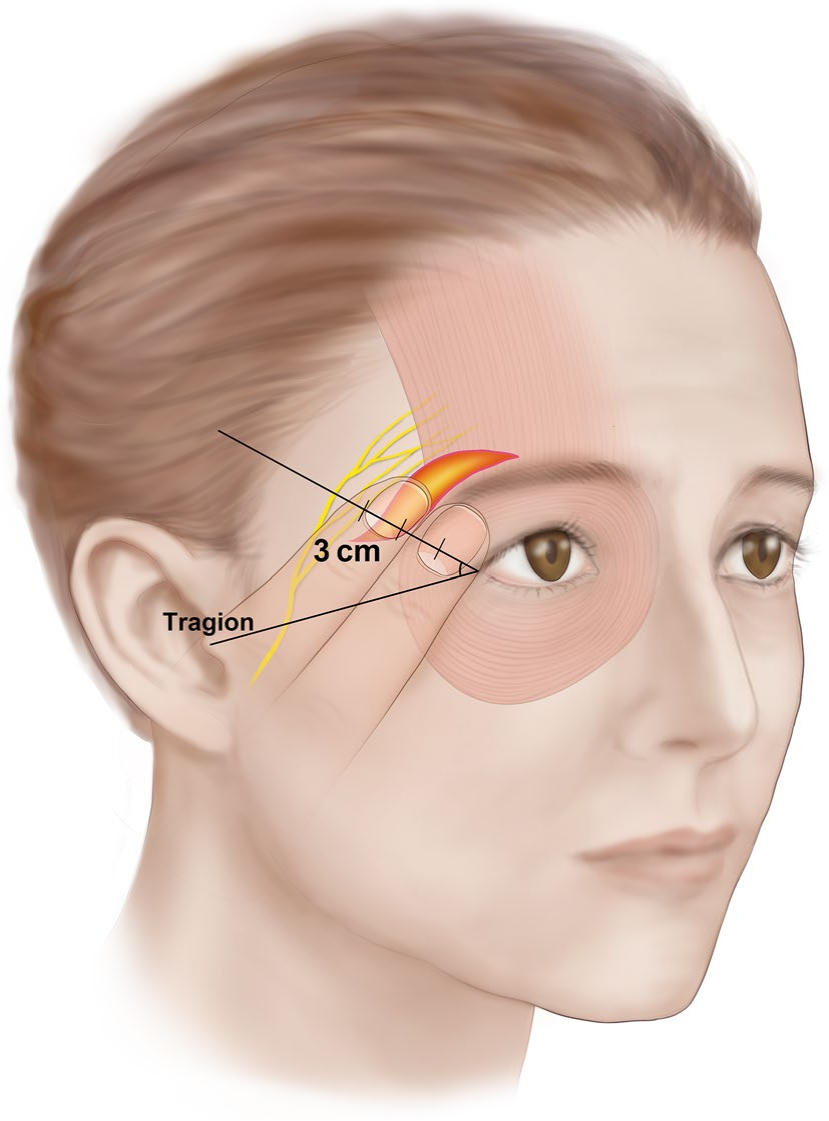
Simple criteria for the positioning of the FbFN during temporal direct browplasty for preventing nerve injury.

Temporal direct browplasty is an excellent procedure for the repair of the lateral third of brow ptosis. The major morbidity associated with temporal brow lift is trauma to FbFN^3, 6^. The general course of this nerve has been documented, but exact details for clinical application in the temporal brow lift are still lacking. This anatomy study was designed to further elucidate its path in the area superotemporal to brow. The results of the present study indicate that the FbFN genernally divide into three branches where they passing though the RL. The crossing points of FbFN-A and FbFN-P were located 3 cm and 4 cm superolaterally from the lateral canthus along with the RL, respectively. Also, the densest area of nerve fibers on the RL being around the 3–4 (mean 3.5) cm section from the lateral canthus. Interestingly, despite inter-individual differences, because the breadth of the fingertip is about 1.5–2 cm, it could also be considered that the FbFN is cross the RL approximately two finger breadths superolaterally from the lateral canthus (Fig. 5).

In the present study, we established the RL in the direction of 45° to the horizontal line connecting the tragion to the lateral canthus from the lateral canthus. Previous studies have reported the usefulness of the MZTV, which is a perforating vessel that passes through the superficial temporal fascia in the superolateral orbital area as an anatomical landmark of FbFN in the temporal region. Its recognition as an important consistent landmark for the conservation of the FbFN in the lateral orbital area has led to the labeling of the MZTV as the sentinel vein. A previous report by Yang et al.^11^ demonstrated that the sentinel vein was located at the lateral portion of the orbicularis oculi about 2 cm superior and 2 cm posterior to the lateral end of the lateral canthus, which is in the direction of 45° from the lateral canthus. Thus, RL in the present study passing through the sentinel vein could be used as a reference line for the determining of the location of FbFN. We also have found that this RL commonly passes through the lateral end of the eyebrow and thus it could be clinically useful during temporal direct brow plasty.

Moreover, the RL could be a good reference line for avoiding the path of FbFN because the RL crosses the previously described ‘red zone’ in the temporal region (red box in Fig. 3). As previously described, the most dangerous area of dissection “red zone” during the temporal lift procedure lies within the 2-cm-wide area superolateral to the brow because the FbFN laying in the innominate fascia (superficial to the deep temporal fascia) is not completely protected under the frontalis muscle and highly vulnerable to iatrogenic injury during the procedure^13^. In all cases the nerve was found to be located in the fascial layer between the superficial and deep temporal fasciae, which results is well correspond with previous anatomical studies^14–16^.

These anatomic observations are useful for the oculofacial surgeon who needs to be aware of how far and deep the dissection can be safely performed during temporal direct browplasty. The FbFN runs on the innominate fascia, crossing the RL at a point approximately two finger breadths superolateral to the lateral canthus. Skin incisions might be placed below this point to avoid nerve injury during the temporal direct browlift. Similarly, dissection should be carried out superficial to the innominate fascial layer which is observed between the superficial temporal fascia and sDTF in the upper temporal compartment to avoid the iatrogenic injury of FbFN (Fig. 3). Simply drawing RL before starting surgery and marking the crossing point (3 cm superolateral to the lateral canthus where the anterior branch of FbFN is located) along this line would help to prevent nerve damage during surgery.

**Figure 6:**
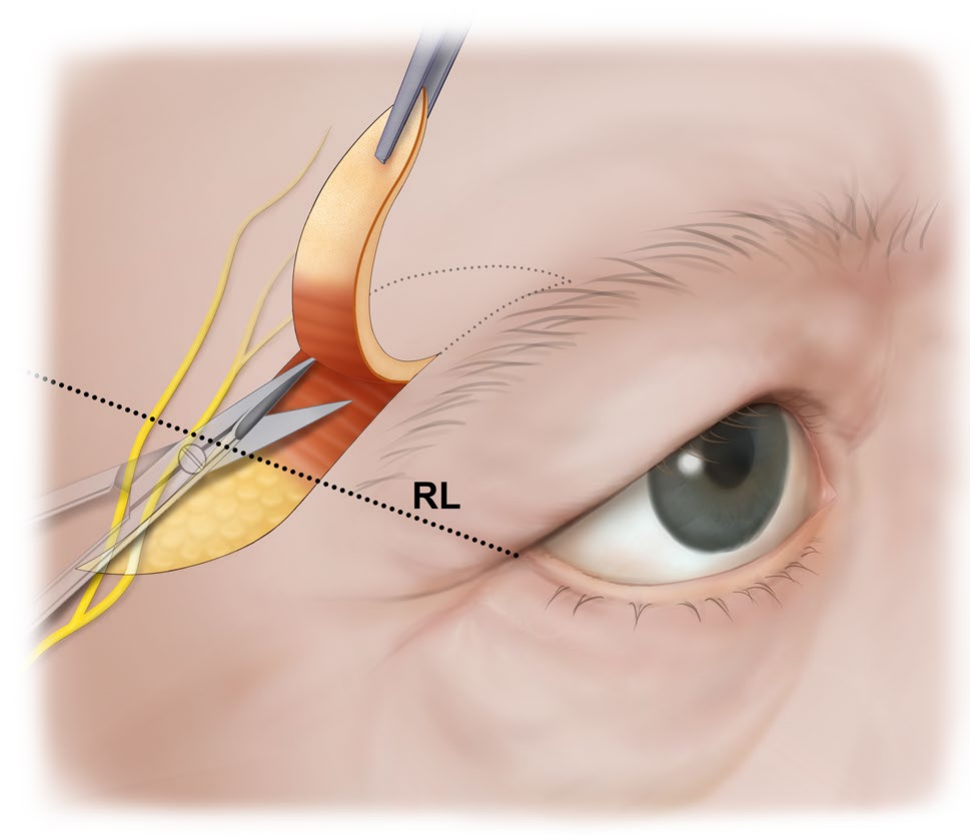
Schematic drawing of temporal direct browplasty. Stay within the subcutaneous fat layer around the RL.

For example, in patients with severe brow ptosis, deep dissection with a large area of tissue excision is necessary. In such cases, great care should be taken not to make the lateral part of the incision too deep near the RL and the oculofacial surgeon must then bring the dissection plane of the forehead tissue more superficially toward its lateral end and stay within the subcutaneous fat layer around the RL, since there is proximal twigs of FbFN and no muscle cushion in this area (Fig. 6)^6^. When this proximal branch is transected, recovery may take year, and sometimes normal frontalis function never return^17^.

Our anatomical findings have been consistent with those of previous reports in respect to the branching pattern of FbFN. Ishikawa^18^ contended that 3 rami of the temporal branch mingled with each other, and the anterior and the middle ramus innervated mostly the frontalis and the superior portion of the orbicularis oculi muscle. Hwang et al.^19^ also investigated the pattern of the FbFN in the frontotemporal region in 20 cadavers. The FbFN was under the superficial temporal fascia above the zygomatic arch and divided into 2 to 4 branches.

Our study group have previously reported the topography of SON in the medial forehead region to avoid its injury during direct browplasty. The vertical line through the lacrimal caruncle provides a convenient guide of the danger zone during the dissection of brow tissue^9^. These collected data for the detailed location of FbFN in the temporal region could be used together in the clinical field for avoiding iatrogenic damage both sensory and motor nerve during direct browplasty.

Additionally, the results of the current study also could be used for preventing iatrogenic facial nerve injury during supraorbital craniotomy through eyebrow incision. The supraorbital craniotomy has been used to access intracranial locating anterior and middle cranial fossae^20^. The supra-eyebrow skin incision runs laterally from the midpupillary line, measuring 4–5 cm in length^21^. To avoid injury to the FbFN, the skin incision is not extended laterally 3 cm from the lateral canthus along the RL where the anterior branch of FbFN is located.

The limitations of the present study included that most of the specimens were from elderly individuals. The nerve position in the temporal area in older patients may differ from that in younger people since the placements of the structures in the orbit typically alter throughout orbit growth in childhood^22^. The results presented here should, however, be representative of those observed in the typical surgical setting given that temporal direct browplasty is more frequently carried out in the older population. Furthermore, topographic variation of FbFN has already been documented^23, 24^. The distance from the lateral canthus to the crossing points of FbFN on the RL can be variable because of individual differences in craniofacial morphology. Therefore, combining Doppler investigation for determining the location of the sentinel vein is a helpful practice to map out the location of the FbFN which is located around the sentinel vein in the individual’s case^25^.

In conclusion, successful temporal direct browplasty for both esthetic and functional purposes requires accurate knowledge of the FbFN course due to its anatomic proximity to the surgical site. Our study has demonstrated that the RL through the lateral canthus provides a convenient guide of the danger zone during the dissection of brow tissue in the temporal region. Placement of the incision within the 3 cm from the lateral canthus along with RL, and avoiding the lateral extension of brow incision beyond the RL, and avoiding deep dissection over the superficial temporal fascia are important in preserving FbFN during the temporal direct browplasty. The detailed topographic information about the FbFN provided by this study may assist oculofacial surgeons in using safer and more effective approaches and help toward improving patient satisfaction.

## Data Availability

All relevant data are included in the manuscript and its table.
